# Polycystic kidney disease strikes a nerve

**DOI:** 10.14814/phy2.15078

**Published:** 2021-10-19

**Authors:** Bo Yang, Steven D. Crowley

**Affiliations:** ^1^ Division of Nephrology & Endocrinology Department of Internal Medicine Naval Medical Center of PLA Second Military Medical University Shanghai People's Republic of China; ^2^ Division of Nephrology Departments of Medicine Durham VA and Duke University Medical Center Durham North Carolina USA

**Keywords:** polycystic kidney disease, sympathetic nervous system

Autosomal dominant polycystic kidney disease (ADPKD) is an inherited condition characterized by the development of numerous fluid‐filled cysts throughout the kidneys. Although it is well established that the dysfunction of polycystin 1 or 2 resulting from mutations of pkd1 or pkd2 is a necessary initiating step for the uncontrolled tubular cell growth and fluid secretion, the pathobiological processes downstream of pkd1 or pkd2 mutations leading to ADPKD still require elucidation. In the original research paper by Giorgia et al. (2021) entitled, “β3 Adrenergic Receptor as a potential therapeutic target in ADPKD,” the authors provide evidence that the activation of the β3 adrenergic receptor (β3 AR), a sympathetic nervous transmitter receptor, may affect the progression of ADPKD through the cAMP signaling cascade in kidney tubular cells.

In these studies, β3 AR expression could be detected in tubular epithelial cells from mice and humans. Moreover, in conditional pkd1 knockout mice and ADPKD patients, β3 AR was upregulated in the kidney (Giorgia et al., 2021). Prior to these studies, β3 AR was found to be expressed in adipose tissue, heart, blood vessels, gall bladder, gastrointestinal tract, prostate and urinary bladder detrusor, brain, and near‐term myometrium (Yang & Tao, 2019). The physiological functions of β3 AR include mediating metabolic, cardiovascular, and nonvascular smooth muscle effects (Grujic et al., 1997; Ursino et al., 2009). However, no previous study to our knowledge has focused on the kidney expression of β3 AR. With immunofluorescence and immunohistochemistry studies, Giorgia et al. suggested that β3 AR localizes within the basolateral plasma membrane of epithelial cells in tubular segments including the thick ascending limb, distal convoluted tubule, and cortical collecting duct (Giorgia et al., 2021). The presence of β3 AR on the epithelial cells would typically indicate sympathetic innervation. To verify the physiological or pathophysiological functions of the sympathetic nervous system through β3 AR in kidney epithelial cells, the demonstration of nerve fibers and nerve endings that connect to epithelial cells will also be required.

Giorgia et al. (2021) illustrate that activation of β3 AR may impact the progression of ADPKD. As additional background for these studies, investigating the sympathetic activity in epithelial cells of normal kidney tubules with its potential downstream effects on cAMP signals would also be of interest. In ADPKD, it is well known that cAMP activity in tubular epithelial cells plays a key role in disease progression (Antignac et al., 2015), and the current studies suggest that β3 AR activation may augment cAMP levels in the kidney epithelium. This finding suggests that sympathetic hyperactivity, which leads to β3 AR activation, could exacerbate the growth of the cysts. We are not aware of clinical studies describing a relationship between sympathetic activity and PKD progression in humans. However, in an observational study of PKD patients with and without hypertension, hypertensive PKD patients had increased muscle sympathetic nerve activity (MSNA) compared with normotensive PKD patients and healthy controls. Nevertheless, no difference in MSNA was detected between normotensive PKD patients and health controls (KLEIN et al., 2001), suggesting that at least in this study, sympathetic tone was not associated specifically with PKD. On the other hand, case reports suggest that renal denervation may be effective for blood pressure control in patients with treatment‐resistant hypertension related to ADPKD that is complicated by CKD (Riccio et al., 2014). Thus, it remains possible that either the sympathetic nervous system regulates blood pressure in ADPKD independently of PKD progression or that the sympathetic nervous system is only engaged during ADPKD once CKD emerges.

The limitations of the current paper should also be considered. The authors suggest that the administration of the β3 AR blocker, SR59230A, may lower renal cAMP level and consequently ameliorate the cystic phenotype (Giorgia et al., 2021). SR59230A is thought to be a selective β3 AR antagonist, but research also suggests it has α1 AR antagonist activity (Bexis & Docherty, 2009). Whether this off‐target effect is a confounding factor warrants scrutiny. An additional question not addressed by the manuscript is how a pkd1/2 mutation engenders hyperactivation of the sympathetic system.

In conclusion, the current manuscript from Giorgia et al. (2021) reveals a potential role of sympathetic nervous system activation in ADPKD progression (Giorgia et al., 2021). The activation of the renal β3 AR may lead to sustained elevation of cAMP levels in kidney epithelial cells and expand cysts. Accordingly, pharmacological blockage of the renal β3 AR could offer potential therapeutic benefit in ADPKD. Future basic studies are needed to provide evidence of direct β3 AR‐mediated sympathetic innervation of tubular epithelial cells. Evidence from clinical studies is also needed to support the notion that hyperactivity of the sympathetic nervous system is an independent risk factor for ADPKD progression.

## CONFLICT OF INTEREST

The authors have no conflict of interest to declare.
